# Convergent evolution, habitat shifts and variable diversification rates in the ovenbird-woodcreeper family (Furnariidae)

**DOI:** 10.1186/1471-2148-9-268

**Published:** 2009-11-21

**Authors:** Martin Irestedt, Jon Fjeldså, Love Dalén, Per GP Ericson

**Affiliations:** 1 Molecular Systematics Laboratory, Swedish Museum of Natural History, PO Box 50007, SE-10405 Stockholm, Sweden; 2 Zoological Museum, University of Copenhagen, Universitetsparken 15, DK-2100 Copenhagen, Denmark; 3 Department of Vertebrate Zoology, Swedish Museum of Natural History, PO Box 50007, SE-10405 Stockholm, Sweden

## Abstract

**Background:**

The Neotropical ovenbird-woodcreeper family (Furnariidae) is an avian group characterized by exceptionally diverse ecomorphological adaptations. For instance, members of the family are known to construct nests of a remarkable variety. This offers a unique opportunity to examine whether changes in nest design, accompanied by expansions into new habitats, facilitates diversification. We present a multi-gene phylogeny and age estimates for the ovenbird-woodcreeper family and use these results to estimate the degree of convergent evolution in both phenotype and habitat utilisation. Furthermore, we discuss whether variation in species richness among ovenbird clades could be explained by differences in clade-specific diversification rates, and whether these rates differ among lineages with different nesting habits. In addition, the systematic positions of some enigmatic ovenbird taxa and the postulated monophyly of some species-rich genera are evaluated.

**Results:**

The phylogenetic results reveal new examples of convergent evolution and show that ovenbirds have independently colonized open habitats at least six times. The calculated age estimates suggest that the ovenbird-woodcreeper family started to diverge at ca 33 Mya, and that the timing of habitat shifts into open environments may be correlated with the aridification of South America during the last 15 My. The results also show that observed large differences in species richness among clades can be explained by a substantial variation in net diversification rates. The synallaxines, which generally are adapted to dry habitats and build exposed vegetative nests, had the highest diversification rate of all major furnariid clades.

**Conclusion:**

Several key features may have played an important role for the radiation and evolution of convergent phenotypes in the ovenbird-woodcreeper family. Our results suggest that changes in nest building strategy and adaptation to novel habitats may have played an important role in a diversification that included multiple radiations into more open and bushy environments. The synallaxines were found to have had a particularly high diversification rate, which may be explained by their ability to build exposed vegetative nests and thus to expand into a variety of novel habitats that emerged during a period of cooling and aridification in South America.

## Background

The New World ovenbirds and woodcreepers have long been recognized as a monophyletic lineage based on a shared unique syrinx structure [1,2]. Until recently most classifications [3-5] have treated ovenbirds and woodcreepers as separate families and subdivided the ovenbirds further into the three subfamilies Furnariinae, Synallaxinae and Philydorinae. This general classification was mainly based on differences in external morphology, which in turn is related to habitat preference and different ways of locomotion and feeding.

As such, the ovenbird-woodcreeper assemblage shares adaptive features across the entire passerine radiation; 1) the ecomorphological variation in Furnariidae encompasses phenotypes that closely matches those of creepers, warblers, wheatears, thrashers, thrushes, bulbuls, dippers, jays, starlings, etc. [6,7], 2) members of Furnariidae have successfully colonized a wide variety of habitats, from the treeless grasslands in the Andes, through humid forests, savanna, and desert, to the coastal shoreline, 3) the variation in nest construction in the family approaches that found across the entire order of passerines [8,9]. Due to this great variation it is not surprising that recent molecular studies [10-17] have revealed several examples of convergent evolution and that the phylogenetic relationships among ovenbirds and woodcreepers are much more complex than suggested in traditional linear classifications.

Among the examples of convergent evolution are the earthcreepers (*Upucerthia *sensu lato) that despite a similar external morphology represent several independent adaptations to terrestrial life in open country [14,17], and the parallel adaptation of *Limnoctites*, *Spartonoica *and *Limnornis/Phleocryptes *lineages to a marsh-dwelling life-style [15]. It is apparent that members of the traditional three ovenbird subfamilies share a functional morphology rather than a close ancestry. Furthermore, it has been demonstrated that woodcreepers represent a specialization for scansorial life within the broader furnariid radiation [10,13].

The extraordinary diversity of adaptations in the ovenbird-woodcreeper family offers a unique opportunity to study the evolution of morphological, ecological and behavioral traits, as well as to explore how adaptations and habitat shifts facilitate further diversification and evolutionary success. Such studies may not only shed light on the evolution of the ovenbird-woodcreeper clade *per se*, but also provide insight into the role of adaptation for the diversification process. So far, a comprehensive evaluation of morphological, ecological and behavioral diversity in the ovenbird-woodcreeper clade has been hampered by the lack both of a well-supported phylogeny that includes all major lineages, and reasonable age estimates of the divergences within this clade.

We present a well supported multi-gene phylogeny that includes more than one third of all species recognized in Furnariidae, including representatives from most genera and multiple species of large and heterogeneous genera. The phylogeny includes representatives of almost all major morphological, ecological and behavioral lineages, and the taxa that are missing mostly belong to species-rich and close-knit genera (e.g., *Geositta*, *Cinclodes*, *Synallaxis*, *Cranioleuca*, *Xiphorhynchus*, *Asthenes *and *Philydor*; some of these cases being covered in other more detailed studies, see [18-22]).

We use the phylogeny and age estimates to study the relationship between major shifts in nest construction and habitat preference with the climatic and ecological history of South America. We also investigate whether the observed variation in species richness between ovenbird clades can be explained by differences in diversification rates among clades. Specifically, we investigate if the diversification rate has been particularly high in the synallaxine clade, since it has been proposed that the change from cavity nests to vegetative nests associated with this clade may have facilitated the colonization of and adaptive radiation within new habitats [12]. Finally, we investigate the systematic position of certain enigmatic ovenbird taxa and evaluate the postulated monophyly of some species-rich genera.

## Methods

### Taxon sampling, amplification and sequencing

The 105 ingroup species in this study represent more than one third of all species in Furnariidae recognized by Remsen [7]. The species selected cover all major radiations of ovenbirds and woodcreepers as suggested by recent molecular studies [10-14,16,17]. We have also included several taxa whose affinities have been difficult to establish by morphology and that had not been included in previous molecular studies, and we have sampled certain genera whose monophyly have been contested more densely [7]. Only five genera (*Gyalophylax*, *Thripophaga*, *Acrobatornis*, *Anabazenops *and *Cichlocolaptes*) out of 69 in the ovenbird-woodcreeper assemblage are not included in the study. Based on their overall morphology, most of these taxa are probably correctly placed in recent classifications and their omission here should only have marginal effect on the results. The only obvious exception is the genus *Thripophaga*, which although presumed to belong to the synallaxine group [7], may not form a monophyletic clade.

To root the phylogenetic trees we included a representative of the sister clade to all other passerines, the New Zealand rifleman, *Acanthisitta chloris*, as well as a parrot and a bee-eater. To break up the long branch between *Acanthisitta chloris *and the ovenbird-woodcreeper assemblage we also included two oscines and 12 additional suboscines.

Five independent loci comprising 3674 bp of DNA have been sequenced and used in the phylogenetic analyses: the nuclear loci glyceraldehyde-3-phosphodehydrogenase (G3PDH) intron 11, myoglobin intron 2, β-fibrinogen intron 5, and ornithine decarboxylase (ODC) introns 6 and 7 (along with the intercepting exon 7), and the mitochondrial cytochrome *b *gene. Extractions, amplifications, and sequencing procedures for fresh tissue/blood samples follow [10,23-25] while corresponding procedures for study skin samples follows [26]. Several new internal primers were designed for the amplification of the cytochrome *b *gene from study skins, Cytb-furnH1 (GTT GTC AAC TGA GAA TCC TCC TCA), Cytb-furnH3 (TCA GAA TGA TAT TTG GCC TCA TGG), Cytb-furnH4 (ARA AGT ATG GGT GGA ATG GGA T), Cytb-furnH5 (ARG TTA TTG TTC GTT GTT TTG AT), Cytb-furnL1 (GTC CTA CCA TGA GGC CAA ATA TC), Cytb-furnL4 (CYY TAG GAA TYT CAT CAA ACT G), Cytb-furnL5 (GCT CTA GCY CTC GCT GCY TCA GT), and Cytb-furnL6 (TAA TAG CAA TAC ACT AYA CAG C).

For each gene and taxon, multiple sequence fragments were obtained by sequencing with different primers. These sequences were assembled to complete sequences with SeqMan II (DNASTAR Inc.). Positions where the nucleotide could not be determined with certainty were coded with the appropriate IUPAC code. Due to the low number of indels in the introns the combined sequences could easily be aligned by eye. All gaps have been treated as missing data in the analyses. No insertions, deletions, stop or nonsense codons were observed in any of the cytochrome *b *sequences, and it therefore seems highly likely that the sequences are of mitochondrial origin and not nuclear copies (numts). Voucher and GenBank accession numbers are given in Additional file 1: Table S1.

### Phylogenetic inference and model selection

We used two model-based methods to estimate phylogenetic relationships; Bayesian inference and maximum-likelihood analysis. The models for nucleotide substitution used in the analyses were selected for each gene individually by using Akaike Information Criterion (AIC,[27]) and the program MrModeltest [28] in conjunction with PAUP* [29]. Bayesian inference was used in the analyses of the concatenated dataset of all genes, a dataset consisting of the nuclear genes only, and the cytochrome b dataset. The posterior probabilities of trees and parameters in the substitution models were approximated with MCMC and Metropolis coupling using the program MrBayes 3.1 [30]. The models selected for the individual gene partitions were used, but the topology was constrained to be the same. Chains were run for 20 million generations with a random starting tree and trees were sampled every 100th generation. The program AWTY [31] was used to estimate when the chains had reached its apparent target distribution and trees sampled during the burn-in phase were discarded.

As the Bayesian analyses from the individual nuclear loci did not converge to the apparent target distribution (due to few variable nucleotides compared to the included number of taxa), these gene trees were estimated using maximum-likelihood analyses in the program GARLI [32].

### Calibration of divergence time estimates and calculation of diversification rates

We used a relaxed clock model implemented in Beast 1.4.7 [33,34] to estimate divergence times between phylogenetic lineages based on the concatenated dataset of all genes. The chains were run with the GTR+I+Γ model for 20 million generations with a random starting tree, and trees were sampled every 1000th generation. As calibration point the geological split between New Zealand and Antarctica was used, as it has been related to the basal separation of the *Acanthisitta*-lineage from the other passerines [35-37]. The dating of this split has often been assumed to be around 85-82 Mya [38], but more recently the timing of this split has been suggested to be more uncertain, 85-65 Mya [39,40]. In order to account for this uncertainty we used a normal distributed tree prior with a median at 78 Mya and a standard deviation of 7 (quantiles 2,5% = 63,3 Mya, 5% = 65,7 Mya, 95% = 90,3 Mya, 97,5%= 92,7 Mya). As for other priors, we used all default settings, except for the Tree Prior category that was set to Yule Process and an uncorrelated lognormal distribution for the molecular clock model [33].

It has been suggested that changes in nest construction strategy have served as an ecological release in furnariids, and particularly in the synallaxine clade [12]. We thus explored the diversification rate for the synallaxine clade and compared it to the diversification rates of other furnariid clades. All species of ovenbirds and woodcreepers recognized by Remsen [7] and Marantz et al. [41] were included in the calculations of diversification rates (except the 4 species of *Thripophaga *whose precise placement within the synallaxine radiation is uncertain, see Material and Methods). The systematic position of species not included in the phylogenetic analysis was assessed based on the relationships suggested by Remsen [7] and Marantz et al. [41].

We generally calculated the diversification rates for clades where all members have the same principal nesting habit, but in a few small clades both cavity and exposed vegetative nesters occur. In a few cases, when information of nesting habits was lacking, we postulated that the species has the same nesting habit as its closest relatives. We used the descriptions of nesting habits provided by Zyskowski and Prum [9] and Remsen [7] to divide all species into either of these two groups. The variation in ovenbird nests sometimes makes this division arbitrary as some species are difficult to assign to either category (i.e. some species that make "improved cavities" may represent transitional stages towards the derived nesting habit). However, as only a few species build nests that are difficult to assign either category, this should only have a marginal effect on the calculations.

To estimate diversification rates, we used the method described by Magallón and Sanderson [42], which uses a stochastic, time-homogeneous birth and death process depending on diversification rate (r) and the relative extinction rate (ϵ). As we wanted to investigate if certain furnariid clades are excessively species rich or species poor we also estimated a 95% confidence interval for the diversification rate based on the mean diversification rate for the entire ovenbird-woodcreeper family (stem group [42]). Cavity-nesting is reconstructed as the plesiomorphic state in the ovenbird-woodcreeper clade (e.g., [9,12,13]). In lineages where shifts in nest design have occurred these shifts may have occurred anywhere along the ancestral branch. To avoid this uncertainty we consequently used a method to calculate the diversification rates for crown groups [42]. As this confidence interval will depend on the relative extinction rate (which is unknown) we calculated confidence intervals for a comparatively high (ϵ = 0.90), intermediate (ϵ = 0.5) and low (ϵ = 0) relative extinction rate. The clades for which diversification rates have been compared, and their principal nesting types and habitat preferences, are given in Table 1.

**Table 1:** Absolute rate of diversification for major ovenbird-woodcreeper clades.

Clade		Number of species	Age	Diversification rates (ϵ = 0)	Diversification rates (ϵ = 0.5)	Diversification rates (ϵ = 0.90)	Nest type	Habitat
**A (Synallaxinae)**		**124**	**19.2**	**0.22**	**0.20**	**0.13**	**EV**	**X, (F)**

*Cranioleuca*/*Metopothrix*		27	10,6	0.24	0.22	0.12	EV	X, F

*Spartonioca*/*Asthenes cactorum*		7	13,6	0.09	0.08	0.03	EV	X(F)

*Synallaxis*/*Certhiaxis*		38	13,4	0.22	0.20	0.11	EV	X

*Asthenes ottonis*/*A dorbignyi*		29	10,8	0.25	0.22	0.12	EV	X

*Anumbius*/*Hellmayrea*		3	16.0	0.03	0.02	0.01	EV	X(F)

*Phacellodomus*		7	7	0.18	0.15	0.06	EV	X, F

*Sylviorthorhynchus/Aphrastura*		13	17,8	0.11	0.09	0.04	EV	X, F

**B (*Upucerthia*- *Phleocryptes*)**		**21**	**15**	**0.16**	**0.14**	**0.07**	**C, (EV)**	**X**

**C (*Furnarius*)**		**6**	**8.3**	**0.13**	**0.11**	**0.04**	**MN, (C)**	**X**

**D (*Tarphonomus*- *Berlepschia*)**		**7**	**21.8**	**0.06**	**0.05**	**0.02**	**C, (EV)**	**X, F**

**E (*Margarornis*- *Premoplex*)**		**6**	**18.9**	**0.06**	**0.05**	**0.02**	**EV**	**F**

**F (Core Philydorinae)**		**42**	**16.3**	**0.19**	**0.17**	**0.10**	**C**	**F**

**G (*Ochetorhynchus*- *X. milleri*)**		**6**	**20.4**	**0.05**	**0.05**	**0.02**	**C, EV**	**X, F**

**H (Dendrocolaptinae - *Xenops*)**		**55**	**27.7**	**0.12**	**0.11**	**0.07**	**C**	**F**

**I (*Geositta*)**		**11**	**14.4**	**0.12**	**0.10**	**0.04**	**C**	**X**

**J (*Sclerurus*)**		**6**	**14.2**	**0.08**	**0.07**	**0.02**	**C**	**F**

**Ovenbird-woodcreeper clade**		**288**	**32.7**	**0.15**	**0.13**	**0.09**		

The absolute rate of diversification have been estimated for clades A - J in absence of extinction (ϵ = 0), intermediate (ϵ = 0.5) and comparatively high (ϵ = 0.90) relative extinction rate. Principle nesting types and habitat preference are indicated by symbols. Nest type symbols; EV = exposed vegetative, C = cavity, and MN = mud nest. Habitat symbols; X = xeric (open and bushy habitats), and F = forest. Symbols placed between parenthesis indicate that occasional member within the clade build this type of nest or occupy this type of habitat. Diversification rates for larger sub-clades nested within the synallaxines (clade A) are given in lower case and normal font.

As we primarily were interested to investigate if the synallaxine clade has an excessively higher rate of diversification compared to the background rate for the Furnariidae, we also calculated diversification rates for larger sub-clades nested within the synallaxines (the sub-clades for which diversification rates have been calculated are given in Table 1). This was done to examine if certain sub-clades nested within the synallaxines may have a particularly great influence on the calculated diversification rate for the entire synallaxine clade.

## Results

### Sequence variation and selection of substitution models

In total, the concatenated dataset of all genes consists of 3674 bp. Except for a few cases where species lack a short segment of a studied gene region, the obtained sequences from the nuclear loci ranged from 667 bp (*Heliobletus*) to 721 bp (*Grallaria*) in the myoglobin intron 2, from 287 bp (*Chamaeza*) to 410 bp (*Margarornis*) in the G3PDH intron 11, from 491 bp (*Corvus*) to 700 bp (*Tityra*) in ODC intron 6 and 7, and between 519 bp (*Acanthisitta*) and 574 bp (*Merops*) in the β-fibrinogen intron 5, while 999 bp were obtained from the mitochondrial cytochrome *b *gene. Most indels observed in the introns were autapomorphic or synapomorphic when mapped onto the tree topology obtained from the Bayesian analyses of the concatenated dataset. A few indels were found to be incongruent with this phylogeny but these were generally found in highly variable gene regions.

**Figure 1:**
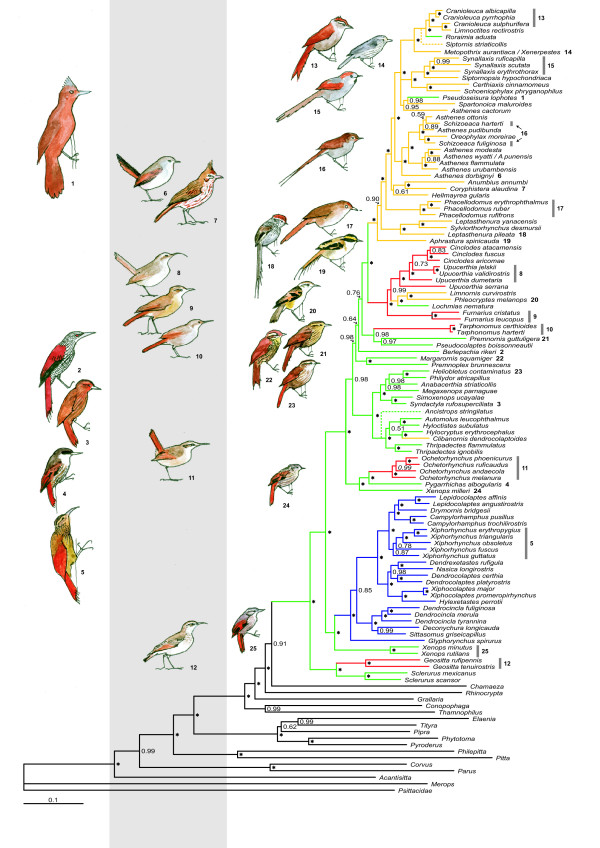
**Phylogenetic relationships of the ovenbird-woodcreeper clade**. The 50% majority rule consensus tree obtained from the analyses of the combined dataset (cytochrome *b*, myoglobin intron 2, ornithine decarboxylase introns 6 and 7, glyceraldehyde-3-phosphate dehydrogenase intron 11, and β-fibrinogen intron 5). Posterior probability values are indicated below the nodes, posterior probability values of 1.00 are indicated with an asterisk. Dashed lines are used to tentatively place taxa (from which nuclear data is lacking) based on their relative position in the cytochrome *b *tree. Differently coloured branches illustrate where the different lineages were placed in traditional classifications (purple = Dendrocolaptidae, green = Philydorinae, red = Furnariinae, yellow = Synallaxinae). The morphological adaptive radiations and examples of convergent evolution within the ovenbird-woodcreeper family are illustrated by drawings to the left of the tree; the phylogenetic positions of the birds are indicated by numbers in bold font. The left column of birds illustrates large scansorial groups (from above; *Pseudoseisura*, *Berlepschia*, *Syndactyla*, *Pygarrhichas*, and *Xiphorhynchus*), the center column depicts groups with terrestrial feeding (from above; *Asthenes dorbignyi*, *Coryphistera*, *Upucerthia*, *Furnarius*, *Tarphonomus*, *Ochetorhynchus*, and *Geositta*), and the right column portrays small acrobatic birds feeding in the vegetation (from above: *Cranioleuca*, *Xenerpestes*, *Synallaxis*, *Schizoeaca*, *Phacellodomus*, *Leptasthenura*, *Aphrastura*, *Phleocryptes*, *Premnornis*, *Margarornis*, *Heliobletus*, *Xenops milleri*, and *Xenops*).

The GTR+I+Γ model of nucleotide substitutions had the best fit for the cytochrome *b *and ODC datasets, while the GTR+Γ model was selected for the myoglobin, G3PDH and β-fibrinogen intron 5 datasets. These models were used in the Bayesian/maximum-likelihood analyses of the individual genes, as well as in the analysis of the combined dataset. The inference for the two concatenated datasets were, after discarding the burn-in phase, based on a total of ca 180,000 samples from the posterior. The posterior distribution of topologies for the concatenated dataset of all genes is presented as a majority-rule consensus tree (Figure 1).

### Phylogenetic relationships

The trees obtained from the maximum-likelihood analyses of the individual gene partitions exhibit different degrees of resolution and are not topologically congruent (Additional file 2: Figure S1). The β-fibrinogen intron 5 produced the topologically most different tree, where, for example, woodcreepers are not recovered as sister group to the core-ovenbirds but are placed at a more terminal position [14]. In general, however, the incongruence is found in parts of the tree with short internodes that have modest support values. Most conflicting topologies are observed in loci with rather few variable positions, where a few homoplasious characters may influence the topology. The cytochrome *b *tree is in general similar to the tree obtained from the concatenated dataset of all nuclear genes (Additional file 2: Figure S1), except that the deeper relationships are poorly resolved. Some conflicts do occur, but mainly at short and poorly supported nodes (the position of the *Xenops minutus *and *Xenops rutilans *lineage is perhaps the most obvious difference between the cytochrome *b *tree and that based on the nuclear genes - see also Fjeldså et al. [13]). However, the overall topology of both the cytochrome *b *tree and the nuclear tree largely agree with the tree obtained from the combined dataset of all genes. No nodes with support values > 0.95 (posterior probability) in the nuclear tree are in conflict with those in the combined tree. The phylogenetic hypotheses are also generally in good agreement with previous phylogenetic studies [10,12-14,16,17,43].

Among the strongly supported relationships, which have not already been demonstrated in other recent molecular studies, are; 1) the affinity of *Xenops milleri *as a basal branch in the *Pygarrhichas*-*Ochetorhychus *clade, instead of being closely related to other *Xenops *species, 2) the position of *Clibanornis dendrocolaptoides *as deeply nested within the core philydorine radiation, rather than being associated with synallaxine ovenbirds as previously assumed, 3) the position of *Sylviorthorhynchus desmursii *as nested within the genus *Leptasthenura*, and 4) the affinity of the small acrobatic/scansorial species *Siptornis striaticollis *and *Roraima adusta *as closely related to the genera *Metopothrix*, *Xenerpestes *and *Cranioleuca*. The phylogenetic results also strongly suggest that the genus *Asthenes *is paraphyletic. *Asthenes cactorum *(and A. humicola [43]) clusters with the genera *Anumbius *and *Coryphistera*, while the other *Asthenes *species (with *A. modesta*, which previously included *A. cactorum *as a subspecies) included in this study form a clade, but with the genus *Schizoeaca *and the monotypic *Oreophylax moreirae *nested among them.

### Divergence time estimates and divergence rates

**Figure 2:**
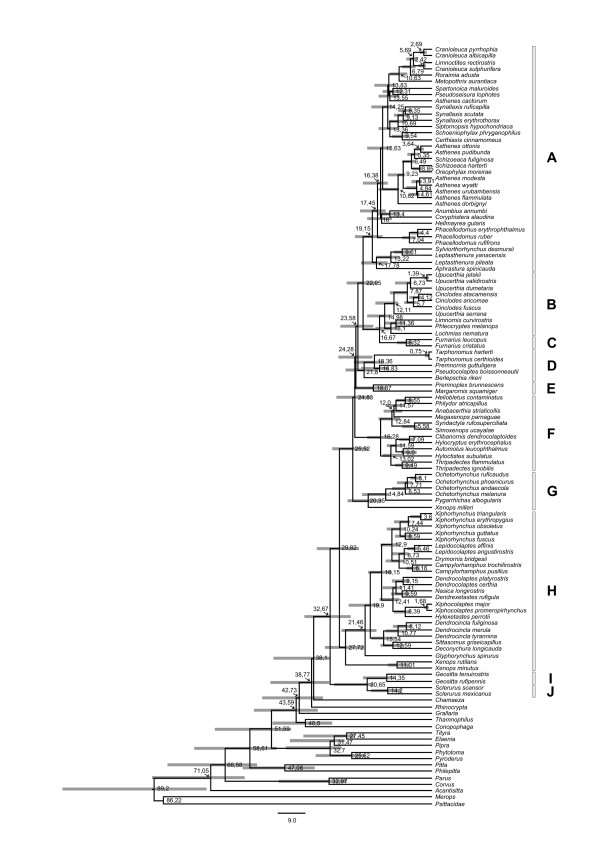
**Chronogram with divergence time estimates of the ovenbird-woodcreeper clade**. The divergence times and confidence intervals (grey bars) were estimated under a relaxed clock model implemented in Beast 1.4.7 [34]. For the calibration of the chronogram the postulated separation of *Acanthisitta *from all other passerines in the phylogeny was used. The letters A - J (on the right) correspond to the clades for which diversification rates have been compared (see Table 2).

Divergence time estimates based on the concatenated dataset suggest that the ovenbird-woodcreeper radiation started to diverge at ca 33 Mya (Figure 2), the time when *Sclerurus *and *Geositta *split off from the core-ovenbird-woodcreeper clade in the Oligocene. The divergence time estimates also suggest that the synallaxines started to diverge at ca 19 Mya, but that most of the generic diversification has occurred during the last 15 My.

**Figure 3:**
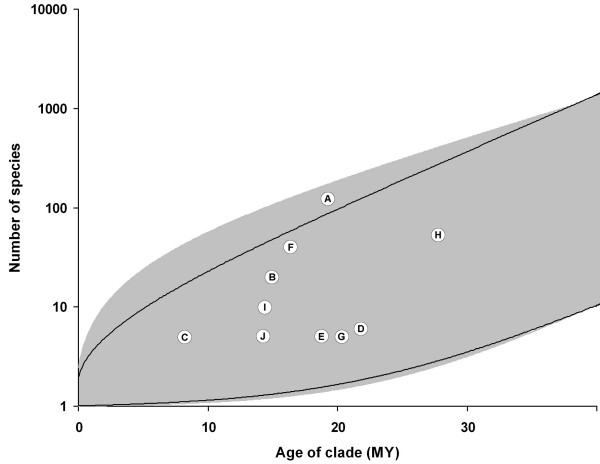
**Plot of diversification rates of major furnariid clades**. The 95% confidence intervals of expected species diversity through time of a clade that diversifies with a rate equal to the ovenbird-woodcreeper radiation as a whole with a relative extinction rate of ϵ = 0.5 (solid line). The shaded area represents the 95% confidence intervals for extreme values of relative extinction rates (ϵ = 0.90; upper boundary, and ϵ = 0; lower boundary). The observed numbers of species in clades A - J (see Table 2) have been plotted against the estimated age of the each clade.

The mean diversification rate for the ovenbird-woodcreeper clade was estimated to 0.15, while the estimated diversification rate for the different sub-clades (A - J in Table 1 and Figure 2) varied between 0.05 and 0.22 (at a relative extinction rate of ϵ = 0). The synallaxine clade was found to have the highest diversification rate (0.22). This rate was significantly higher than the mean diversification rate for the overall ovenbird-woodcreeper lineage at relative extinction rates between 0 and 0.75 (Figure 3). The calculated diversification rates for sub-clades nested within the synallaxines show that there is a variation in diversification rates among the sub-clades (Table 1). However, a majority of the sub-clades (both terminal and more basal clades) have higher diversification rates than the background rate for the Furnariidae at both low and intermediate extinction rates.

## Discussion

### The phylogeny of the ovenbird-woodcreeper assemblage - an extraordinary example of adaptive radiation

The analysis resulted in a well resolved and generally strongly supported phylogeny. Topological conflicts between the different gene trees, as well as between the combined tree and previously published molecular phylogenies almost exclusively concern short internodes with weak support. In some loci with rather few variable positions (e.g., β-fibrinogen intron 5), homoplasious characters may have a significant effect on the tree topology [14]. It is difficult, however, to ascertain whether this is an effect of real biological processes [44] or analytical factors [45-47]. It may thus be assumed that a phylogeny based on DNA sequences from multiple genes provides a better estimate than does a single gene tree. Arguably, the phylogeny presented here is therefore the best estimate of relationships in the ovenbird-woodcreeper assemblage to date, as it includes data from five independent loci and more taxa than previous studies. Our assumption is supported by the observations that: 1) there is a general congruence between the well supported parts of the gene trees, 2) there is a good congruence between the mitochondrial tree and the tree obtained from the concatenated nuclear dataset, and 3) the results are in good agreement with other studies utilizing independent molecular markers [16,17].

The phylogenetic tree obtained from the combined dataset (Figure 1) supports the major phylogenetic relationships demonstrated in previous molecular studies [10-14,16,17,43], including that woodcreepers are nested among ovenbirds, that the traditional subdivision of ovenbirds to a large degree reflects functional groups rather than monophyletic groups, and that the most basal taxa are terrestrial (*Sclerurus *and *Geositta*), which is a trait shared with the closest relatives of the entire ovenbird-woodcreeper radiation (see below). The results also reveal several new examples where the traditional classifications have been misled by convergently evolved morphological characters. The major patterns of diversification in the ovenbird-woodcreeper clade now seem well corroborated, although a denser taxon sampling is needed to fully resolve generic delimitations and to test the monophyly of some larger genera. The phylogenetic hypothesis presented covers most major ecological and morphological shifts in the group, and it is unlikely that the few genera not included in the analysis would change the conclusions below.

The ovenbird-woodcreeper family constitutes one of the best study cases of adaptive radiations in passerines with several examples of convergent evolution in morphology and behavior. Convergence may lead to erroneous conclusions about systematic relationships. This has certainly been the case with the furnariids, as evident from the fact that the long accepted subfamilies of ovenbirds, Furnariinae, Synallaxinae and Philydorinae, are not recovered as reciprocally monophyletic (Figure 1).

The nearest relatives of ovenbirds, tapaculos (Rhinocryptidae) and ground antbirds (Grallaridae, Formicaridae) are ground-feeding forest birds, and it is therefore plausible that the genus *Sclerurus*, which finds its food on the forest floor by flicking away dead leaves, represents an ancestral ecological niche in the Furnariidae. It seems plausible to assume that *Geositta *adapted to tree-less landscapes in response to the aridification that took place in the southern part of the continent from the early Miocene. However, all other early furnariid groups adopted scansorial habitats, as they forage on tree-trunks and in vine-tangles, mainly in evergreen forest habitats, specializing on different sections and kinds of tree trunks [12,13]. The highest diversity of early furnariids is found in the humid tropics, although some deep branches in the temporal austral rainforest (*Pygarrhicas*, *Aphrastura*) suggest a wider distribution at some point during the mid-Tertiary, when Patagonia had more extensive and biologically rich forests [48]. The later radiation in the tropics may to a large extent be linked with the habitat complexity of the Amazon basin and the many marine incursions and formation of various kinds of swamp forests [49], but some members of these scansorial groups became widespread generalists and some switched to drier woodlands.

Within the ovenbird-woodcreeper family, open habitats have been colonized independently at least six times; by *Geositta *(see above), *Drymornis bridgesii *[11], *Ochetorhynchus *(sensu [14]), *Tarphonomus *(sensu [50]), *Clibanornis dendrocolaptoides*, the modified Furnariinae lineage (*Cinclodes *to *Furnarius *in Figure 1) and several synallaxines (especially *Coryphistera*, *Anumbius *and some *Asthenes *species). Among the examples of shifts in habitat utilization revealed by the present study is the Canebrake groundcreeper, *Clibanornis dendrocolaptoides*. This species has formerly been associated with the Furnariinae genus *Cinclodes*, but in recent classifications it is often placed near the Synallaxinae genus *Phacellodomus *[7]. Our results strongly support that *Clibanornis *is deeply nested among core-philydorine ovenbirds. *Clibanornis *feeds on or near the ground, a near unique habit among philydorine ovenbirds, and previous classifications have thus been deceived by its ecological and morphological modifications to this habit.

A particularly interesting example, discussed by Fjeldså et al. [13], is now made more complete by the inclusion of *Xenops (Microxenops) milleri*, which is a rare and poorly known canopy bird of the Amazonian lowland forest. We must assume that its scansorial habits, along with those of the austral *Pygarrhicas albogularis*, represent ancestral habits, and that the *Ochetorhynchus *species, which inhabit the intervening geographical areas in the Andes, diverged and became terrestrial, as they adapted perfectly to the harsh and treeless landscapes that arose as a consequence of Andean uplift and aridification. Thus they became superficially similar to the *Upucerthia *earthcreepers (in the furnariine group), and were until recently included in that group.

The synallaxine group has undergone complex habitat changes. The most basal species inhabit forest (*Aphrastura *in the austral beech forest, *Leptasthenura setaria *in the Brazilian auricaria forests), more open woodlands or bushy habitats in the southern cone of the continent and in the Andes (other *Leptasthenura *species), as well as humid undergrowth in the austral forest zone (*Sylviorthorhynchus desmursii*). The latter species has been assumed to be related to *Schizoeaca *thistletails based on general morphology, decomposed tail structure and nest type [7]. Gonzales and Wink [43] recently provided molecular evidence for placing it near *Leptasthenura*, and our data demonstrates that it is actually nested within that genus. In fact, its dome-shaped nest is similar in construction to that of *Leptasthenura yanacensis *and *fuliginiceps *(JF, unpublished), and unlike other *Leptasthenura *species that nest in somewhat improved or lined holes, or in abandoned nests of other furnarids. Other synallaxines have become adapted to forage in the structurally complex and microphyllic scrubby vegetation in drier habitats in the southern regions and in the Andes, where in some cases they switched to terrestrial feeding. Many species in the Andean zone and Patagonia adapted to shrub steppe and grasslands (*Asthenes*, *Schizoeaca *and *Oreophylax*). In addition, there are also examples of recolonization of humid forests habitats. This is most pronounced within the *Metopothrix*-*Cranioleuca *clade. *Roraimia adusta*, which is confidently placed within this clade, has in fact traditionally been assumed to be related to the small and acrobatic Philydorinae genera *Premnornis*, *Premnoplex *and *Margarornis *[51].

### Rates of diversification and nest habits

Among the "key" features that have been proposed as important for diversification among birds are small body size and short generation time [52], intense sexual selection [53], the ability to build nests of many different types [54], and behavioral flexibility [55]. Several of these features vary significantly among the ovenbirds and it is reasonable to assume that several of them may have influenced diversification rates in the ovenbird-woodcreeper clade. In addition to these factors, it can be assumed that the climatic and ecological development of South America during this time has had an effect on diversification rates.

For the ovenbird-woodcreeper clade, changes in the bill kinesis has been proposed to play a significant role for the earliest diversification of the group. Particularly the unique, flexible pseudo-rhynchokinetic bill is likely to have been important for the evolution of substrate-oriented specializations [13]. The synallaxine radiation is by far the most species rich within the ovenbird-woodcreeper lineage. Irestedt et al. [12] proposed that a shift in nest habit, from cavity-nesting to the ability to build exposed vegetative nests, served as an ecological release that facilitated the diversification of the synallaxines into more open habitats.

The temporal variation in diversification rates is reflected by the variation in branch lengths in the chronogram. Recent studies (e.g., [56]) have shown that it may also be possible to estimate the relative contribution of speciation and extinction to the overall diversification rate. However, this would require taxonomically almost complete phylogenies to avoid that biased taxon sampling obscures the pattern. Our phylogeny lacks too many taxa to make such a study meaningful, as variation in branch lengths in our phylogeny in several cases may reflect incomplete sampling. Instead, we estimated the diversification as described by Magallón & Sanderson [42], which assumes that clades can be treated as independent data points. Our diversification rate estimates show that the synallaxine group has the highest diversification rate of all major furnariid clades (Table 1). This rate is significantly higher than the mean diversification rate for the overall ovenbird-woodcreeper clade assuming low to high (ϵ < 0.75) relative extinction rates (Figure 3). For higher relative extinction rates (ϵ > 0.75), the species richness in the synallaxines lies within the 95% confidence interval of the mean diversification rate. Although the relative extinction rate in the ovenbird-woodcreeper clade currently is unknown, it should be noted that the upper limit used here (ϵ = 0.9) could be considered high [42]. The diversification rate in the synallaxines can therefore be considered as excessively high under most plausible scenarios. None of the other groups were found to be exceptionally species rich or species poor (Figure 3). The calculated diversification rates for sub-clades nested within the synallaxines suggest there is a variation in diversification rates within the synallaxines. However, as several sub-clades (both terminal and more basal clades) have higher diversification rates than the background rate for the Furnariidae, the observed high diversification for the synallaxines is not driven by a particular sub-clade nested within the synallaxines.

By comparing the age estimates calculated in this study (Figure 2) with the climatic and ecological development of South America, it is evident that the radiation of synallaxine ovenbirds mainly took place during the last 15 Mya, which was a period when South America experienced a period of cooling and aridification [57,58]. It is notable that a similar large-scale diversification into open habitats during that time also has been suggested to have occurred within tyrannidae [59]. Cavity-nesting birds may be constrained by a paucity of potential nesting holes in areas with dense shrub steppe habitats (and lack of larger trees, or exposed rocky areas). This should also be the case in the riverine thickets and marsh vegetation, and in the dense vegetation of stunted cloud-forest and paramo shrubbery that developed during the Neogene uplift in the tropical Andes region. The ability, and flexibility, in building vegetative nests in the dense, bushy vegetation may have allowed the synallaxines to build up large populations in such habitats and thus to expand and diversify [60].

The ability to build exposed vegetative nests also occurs in a few species outside the synallaxines, many of which also inhabit open environments. However, these species are nested within clades B, D and G, where cavity-nesting is pleisomorphic, and the ability to build exposed vegetative nests may thus be recently derived. The only other clade where exposed vegetative nest building is synapomorphic is clade E. In contrast to the synallaxines, this clade has a relatively low diversification rate (Table 1). One possible explanation for this is that clade E is confined to a forest environment, which may have restricted its potential to diversify.

Both environmental and behavioral factors constitute morphological constraints on birds, as exemplified by the many similarities in external morphology between various ovenbird-woodcreeper lineages and other passerines. That this "morphological drift" could be rather fast and pronounced in passerines when new habitats or behavioral niches are colonized is evident from the many examples revealed within the ovenbird-woodcreeper radiation, as well as in other passerine birds, e.g., *Pseudopodoces humilis *[61].

## Conclusion

The major patterns of diversification in the ovenbird-woodcreeper clade now seems well corroborated, as our results largely support the phylogenetic relationships demonstrated in previous molecular studies. Our results also reveal additional examples of convergent evolution, suggest that some large genera are not monophyletic and, using molecular methods, place some enigmatic furnariids for the first time. The phylogenetic results further suggest that the ovenbird-woodcreeper clade has independently colonized open habitats several times and that the diversification of clades adapted to open habitat coincides with the aridification of South America. Although several key features may have played an important role for the exceptional adaptive radiation of the ovenbird-woodcreeper family, the observation that the synallaxine clade has the highest diversification rate of all furnariid clades suggests that the shift from a cavity-nesting habit to building more flexible vegetative nests, combined with an expansion into more open and bushy habitats, may have played an important role in the diversification of this group. The results manifest the ovenbird-woodcreeper assemblage as one of the most remarkable examples of adaptive radiation and convergent evolution in passerine birds.

## Authors' contributions

MI designed the study, carried out the labwork, performed the phylogenetic analyses, and drafted the manuscript. JF assisted with the design of the study and with the draft of the manuscript. LD assisted with the diversification analyses and with writing the manuscript. PE assisted with the phylogenetic analyses and conceived the study. All authors read and approved the manuscript.

## Supplementary Material

Additional file 1 **Sample used in the study**. Abbreviations: AMNH = American Museum of Natural History, New York; ANSP = Academy of Natural Sciences of Philadelphia; FMNH = Field Museum of Natural History, Chicago; LSUMZ = Museum of Natural Science, Louisiana State University; MNHN = Muséum National d' Histoire Naturelle, Paris; NRM = Swedish Museum of Natural History, Stockholm; USNM = US National Museum, Smithsonian Institution, Washington DC; ZMUC = Zoological Museum of the University of Copenhagen. Specimens for which no voucher exists are marked with an asterisk. Click here for file

Additional file 2 **Phylogenetic trees based on individual gene partitions**. Phylogenetic trees; A = the 50% majority rule consensus tree obtained from the Bayesian analysis of cytochrome *b *gene (posterior probability values are indicated above the nodes, posterior probability values of 1.00 are indicated with an asterisk), B = the 50% majority rule consensus tree obtained from the Bayesian analysis of the concatenated dataset of all nuclear genes (myoglobin intron 2, glyceraldehyde-3-phosphate dehydrogenase intron 11, ornithine decarboxylase introns 6 and 7, and β-fibrinogen intron 5), C = the tree obtained from maximum-likelihood analysis of myoglobin intron 2, D = the tree obtained from maximum-likelihood analysis of glyceraldehyde-3-phosphate dehydrogenase intron 11, E = the tree obtained from maximum-likelihood analysis of ornithine decarboxylase introns 6 and 7, and F = the tree obtained from maximum-likelihood analysis of β-fibrinogen intron 5. Click here for file
