# Supplementary figures and images for: Convergent evolution, habitat shifts and variable diversification rates in the ovenbird-woodcreeper family (Furnariidae)
Source: BMC Evol Biol. 2009 Nov 21;9:268. doi: 10.1186/1471-2148-9-268 (PMC2787525; doi:10.1186/1471-2148-9-268)

# A

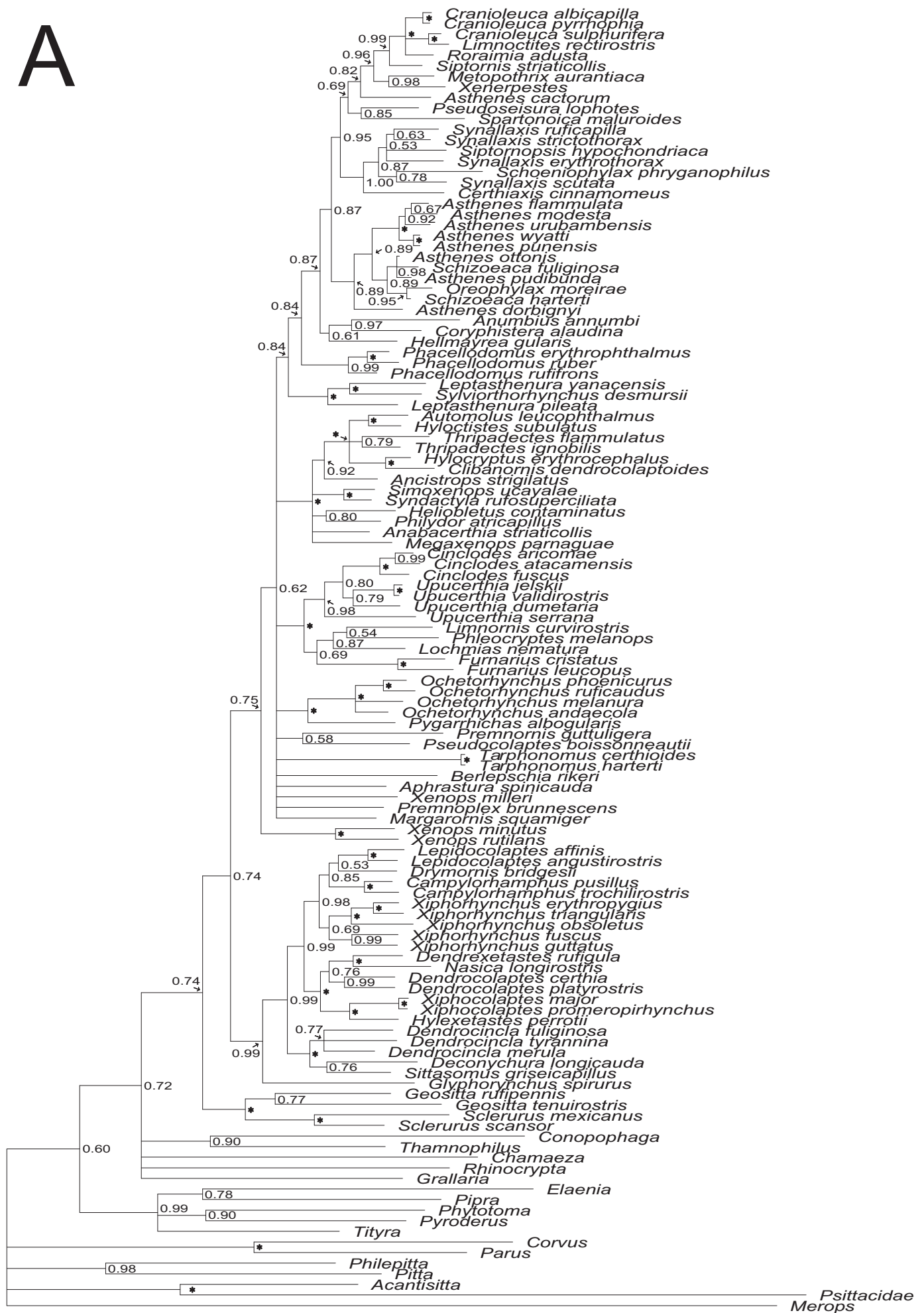

B

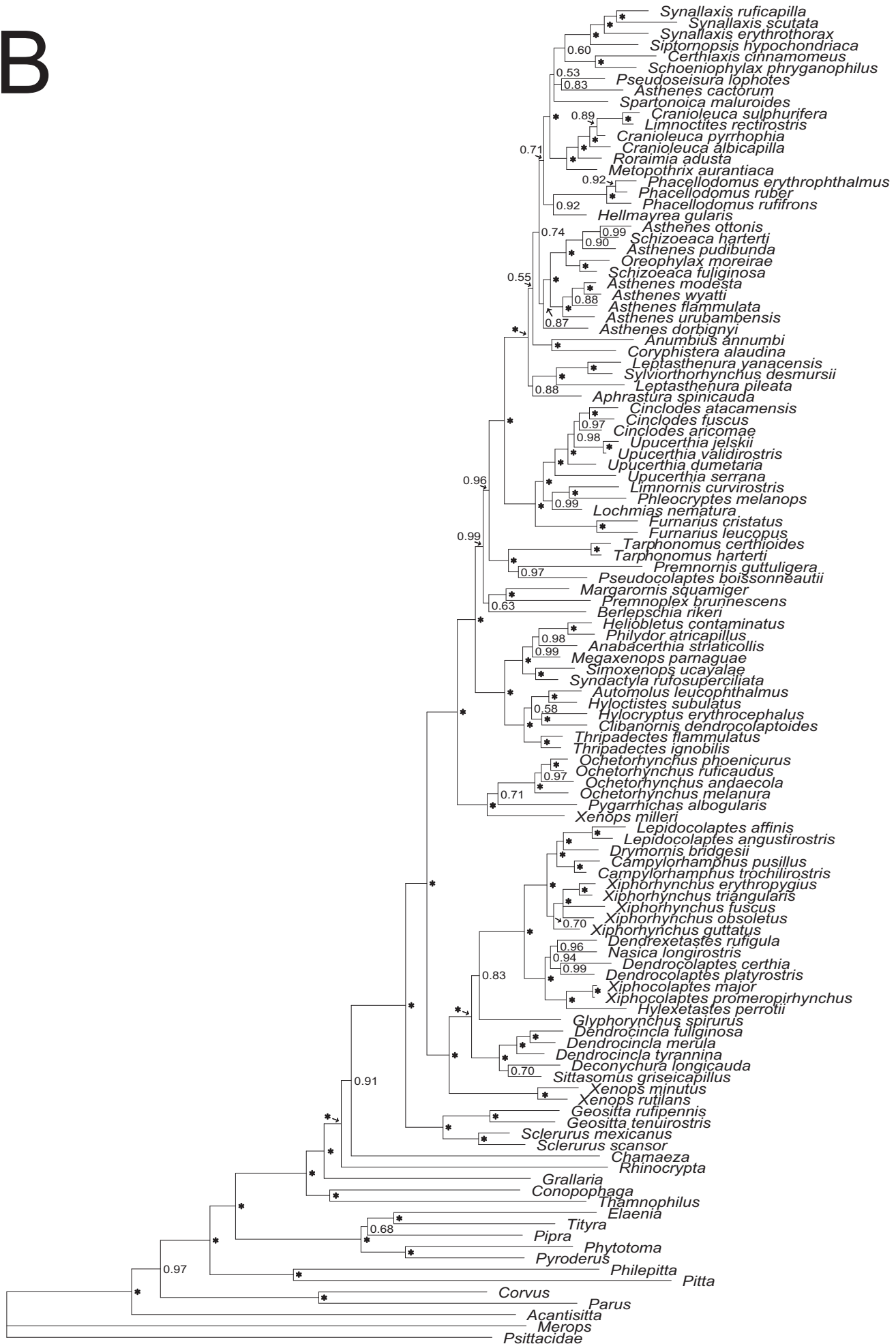

0.1

C

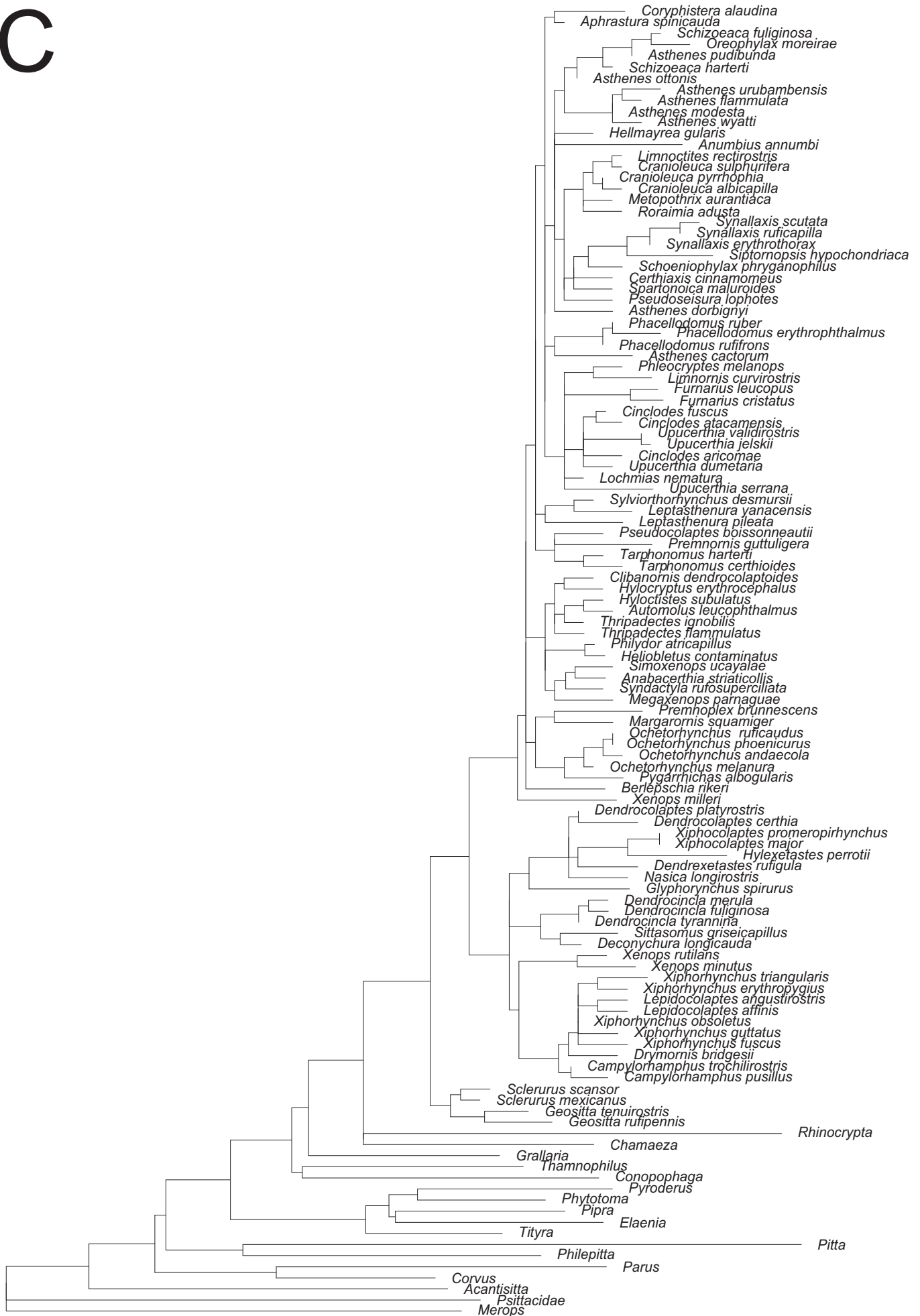

0.1

D

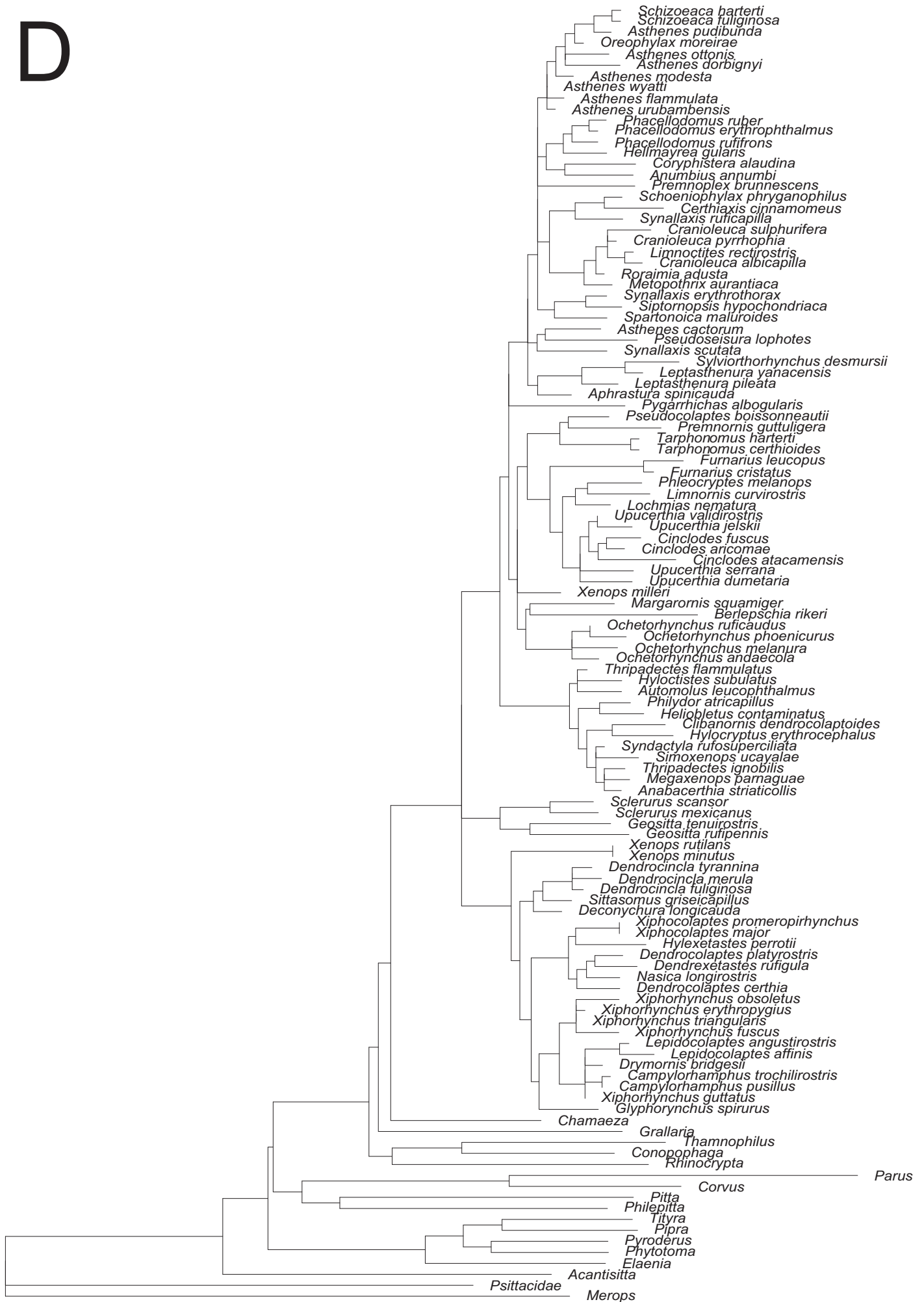

0.1

E

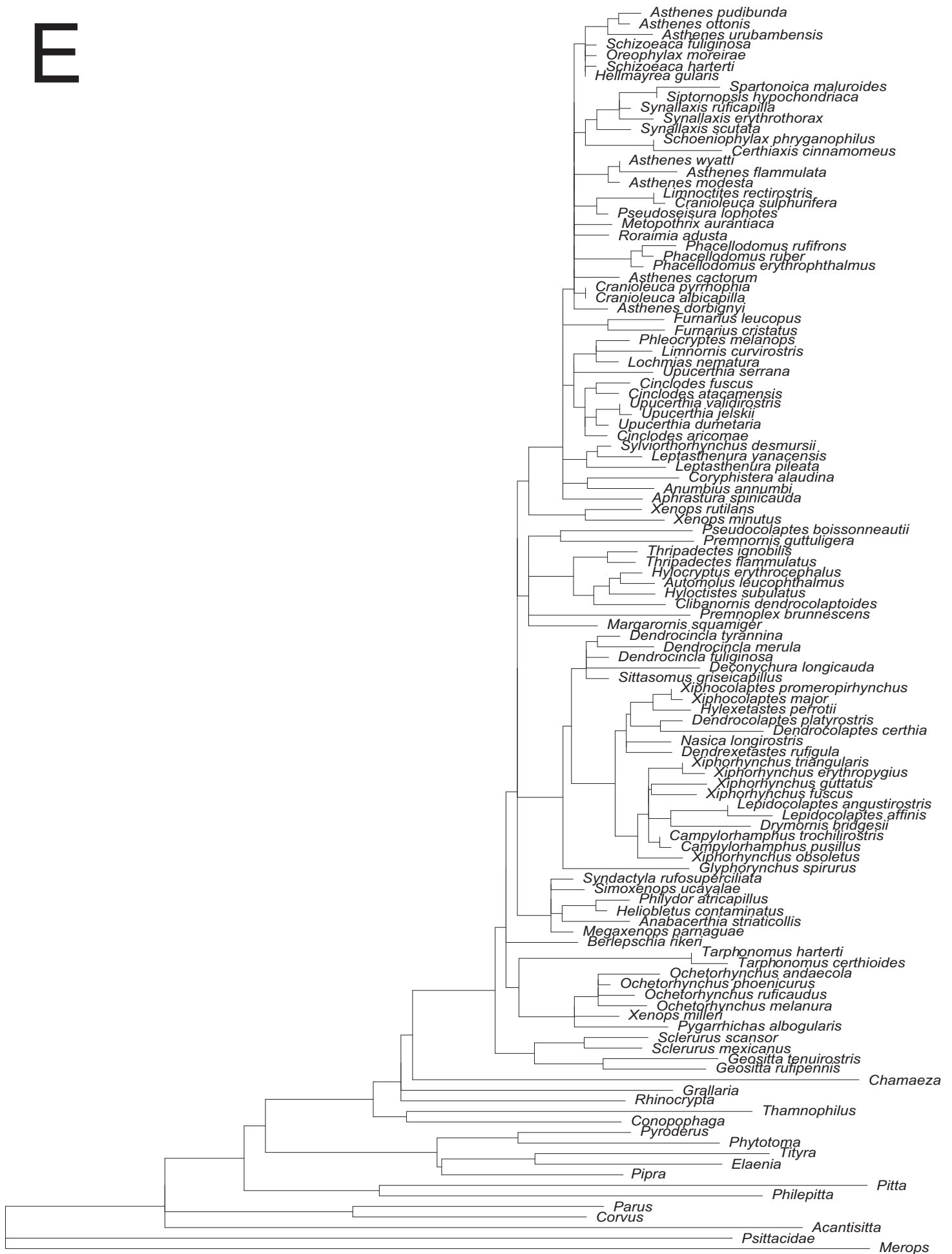

0.1

F

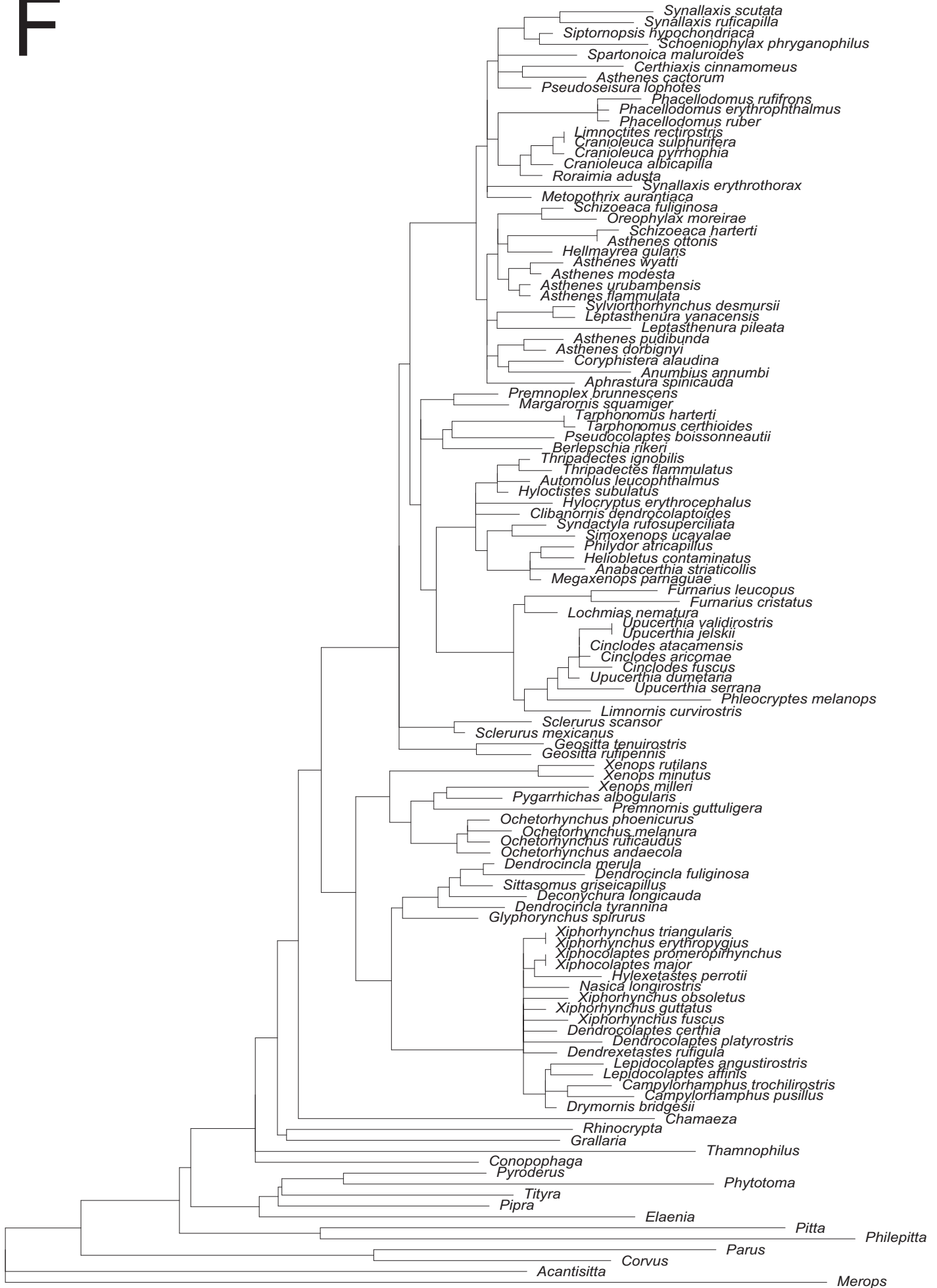

0.1

Supplement: Additional file 2 — Phylogenetic trees based on individual gene partitions. Phylogenetic trees; A = the 50% majority rule consensus tree obtained from the Bayesian analysis of cytochrome b gene (posterior probability values are indicated above the nodes, posterior probability values of 1.00 are indicated with an asterisk), B = the 50% majority rule consensus tree obtained from the Bayesian analysis of the concatenated dataset of all nuclear genes (myoglobin intron 2, glyceraldehyde-3-phosphate dehydrogenase intron 11, ornithine decarboxylase introns 6 and 7, and β-fibrinogen intron 5), C = the tree obtained from maximum-likelihood analysis of myoglobin intron 2, D = the tree obtained from maximum-likelihood analysis of glyceraldehyde-3-phosphate dehydrogenase intron 11, E = the tree obtained from maximum-likelihood analysis of ornithine decarboxylase introns 6 and 7, and F = the tree obtained from maximum-likelihood analysis of β-fibrinogen intron 5. [file 1471-2148-9-268-S2.PDF]
